# Investigating the epigenetic landscape of symptomatic disk degeneration: a case study

**DOI:** 10.1097/PR9.0000000000001237

**Published:** 2025-02-21

**Authors:** Taylor D. Yeater, Yuya Kawarai, Seunghwan Lee, Kumar G. Belani, David S. Beebe, Dmitriy Sheyn, Manuel R. Pinto, Laura S. Stone

**Affiliations:** a Department of Anesthesiology, School of Medicine, University of Minnesota, Minneapolis, MN, USA; b Department of Orthopedic Surgery, Chiba University, Chiba, Japan; c Orthopaedic Stem Cell Research Laboratory, Cedars-Sinai Medical Center, Los Angeles, CA, USA; d Twin Cities Spine Center, Minneapolis, MN, USA

**Keywords:** DNA methylation, Intervertebral disks, Spine, Back pain, Discography

## Abstract

**Introduction:**

This study investigates the epigenetic landscape underlying painful intervertebral disk (IVD) degeneration in a single subject with a history of low back pain (LBP). Intervertebral disk degeneration is associated with LBP in some individuals; however, there is often a discrepancy between degeneration and pain. We hypothesize that DNA methylation, an epigenetic mechanism previously linked to discogenic LBP, is dysregulated in symptomatic vs asymptomatic IVDs.

**Objectives:**

Identify differentially methylated genes and pathways in symptomatic vs asymptomatic IVDs.

**Methods:**

Three lumbar IVDs with similar degeneration severity were tested prior to surgery by discography to identify symptomatic IVDs. Methylation analysis was performed on ∼935,000 cytosine guanine dinucleotide sites on nucleus pulposus DNA. We explored differential methylation and pathway enrichment on cytosine guanine dinucleotide sites located within the promoter regions of genes.

**Results:**

Two IVDs (L3/L4 and L4/L5) evoked pain ratings of 10/10 and 8/10, one IVD (L5/S1) scored 0/10. DNA methylation differed between symptomatic and asymptomatic IVDs. Several identified genes have roles in extracellular matrix remodeling. Other differentially methylated genes were related to immunomodulation and ion channel function. Finally, several long noncoding RNA genes were identified, encouraging further exploration into these regulatory molecules. Enriched pathways were associated with immune response, hormonal regulation, nervous system development, and musculoskeletal development and remodeling.

**Conclusion:**

This case study provides a promising list of candidate genes for therapeutic development for discogenic LBP and suggests a role for DNA methylation in the development of symptomatic vs asymptomatic IVD degeneration, calling for further research to validate and expand these findings.

## Abstract 2

Supplemental Digital Content is Available in the Text.

Differences in DNA methylation were identified in asymptomatic vs symptomatic lumbar intervertebral disks with similar degeneration severity obtained from a single participant with back pain.

## 1. Introduction

Low back pain (LBP) is a leading cause of global disability,^15,42^ with an estimated 40% of all LBP linked to intervertebral disk (IVD) degeneration, referred to as discogenic LBP.^11,24,38^ However, the discordance between structural abnormalities and symptomatic pain remains poorly understood. Numerous studies have highlighted the limited correlation between radiological evidence of IVD degeneration and the experience of LBP. In a systematic literature review, spinal degeneration is clearly present in asymptomatic individuals, with prevalence increasing with age.^8,9^ Studies such as these have challenged the traditional assumption that structural abnormalities per se are the source of LBP, highlighting the need to parse out the complex relationship between disk degeneration and discogenic pain. As highlighted in prior work, this discrepancy may be because the symptoms arise from cellular or molecular changes, such as in gene regulation and expression, that accompany IVD degeneration but are not detectible by imaging.^22,26,29^ This incongruence prompted our case study of a single human subject, focusing on the molecular underpinnings of disk degeneration and its association with symptomatic LBP.

Epigenetic modifications alter the function of DNA without altering the underlying genetic code. One epigenetic modification is DNA methylation, or the addition of a methyl group to a cytosine guanine dinucleotide (CpG). Increased methylation in promoter regions generally leads to a decrease in the expression of the gene. Epigenetic alterations can occur due to environmental factors, aging, diet, exercise, and stress to name a few.^1,13,23,35^ Importantly, these changes are reversible, making them an attractive therapeutic target. In fact, epigenetic reprogramming has been used to reverse aging in mice.^43^

DNA methylation has been implicated in the progression of IVD degeneration.^28^ For example, differences in methylation have been reported in degenerating rat IVDs^19^ and in the promoter region of an extracellular matrix gene in both mouse and human degenerating IVDs.^41^ In addition, blood from humans experiencing LBP or other musculoskeletal pain conditions exhibit differential methylation profiles compared to controls.^4–7,16,17,32,33^ Taken together, these findings suggest that epigenetic alterations play a pivotal role in the initiation and progression of disk degeneration and in the experience of LBP.

Despite these advances, a critical knowledge gap persists in understanding if specific epigenetic signatures are associated with painful compared to asymptomatic disk degeneration. The goal of this case study was to investigate the epigenetic landscape of 3 degenerating IVDs obtained from a single subject—comparing 2 pain-generating IVDs to one nonpainful IVD. This study provides insights into the molecular basis of symptomatic disk degeneration to identify potential therapeutic targets for future study.

## 2. Methods

### 2.1. Experimental design

The samples used in this study were obtained as part of a larger study investigating the molecular underpinnings underlying the link between disk degeneration and LBP.^26,29^ The current study centers on a singular participant exhibiting LBP and pronounced degeneration across multiple lumbar IVDs, necessitating surgical intervention. Prior to surgery, lumbar discography was performed to identify IVDs responsible for pain symptoms (n = 2 painful, n = 1 nonpainful). Nucleus pulposus tissue was collected during surgery and stored at −80°C until processing for DNA methylation. Comparisons are made between the painful and nonpainful degenerating disks.

### 2.2. Subject consent and Screening

Prior to participation, the subject underwent a comprehensive informed consent process. Pain and disability were measured using the McGill Pain Questionnaire^30,31^ and the Oswestry Disability Index,^12^ respectively. Patient history, including use of pain medication, was assessed as previously described.^26,29^ All procedures were approved by the Institutional Review Boards of the University of Minnesota (Protocol #0407 M62061 & STUDY00010758) and Allina Health Hospitals & Clinics (Protocol #1885).

### 2.3. Lumbar discography

Discography is a diagnostic procedure used to identify and assess the source of back pain when it is suspected to originate from the IVDs. The procedure involves the injection of contrast dye into one or more spinal disks to evaluate their structure and detect abnormalities. The injection of this dye also increases the pressure within the disk, potentially triggering recapitulation of the patient's pain experience. During the procedure, the patient is asked to provide feedback about any pain or discomfort experienced using the 0 to 10 numeric rating scale. This information helps the physician correlate the patient's pain symptoms with the appearance of the disk on imaging.

### 2.4. DNA methylation array

Nucleus pulposus samples (50–100 mg) were pulverized in liquid nitrogen with a mortar and pestle and hammer. The resulting fine powder was processed with the Qiagen DNA Mini Kit (Qiagen, Hilden, Germany) using the manufacturer's instructions with the following adjustments: 360 µl of lysis buffer plus 20 μL proteinase k overnight. Nanodrop was used to assess the purity and concentration of DNA in samples. Samples with concentrations < 10 ng/µl or with 260/280 outside of 1.7 to 1.9 were reprocessed using the Zymo DNA Clean (Zymo Research, Irvine, CA) and Concentrate kit. Multiple rounds of DNA extraction from disk tissue were needed to acquire enough DNA (>250 ng) for the methylation assay. The Illumina Infinium MethylationEPIC v2.0 Beadchip was used to investigate over 935,000 CpG sites.

### 2.5. Data analysis

Analysis of the DNA methylation data was executed through the SEgmentation-based State Analysis of Methylation data (SeSAMe) package in *R* (version 4.3.1).^47^ Preprocessing was performed on the data per recommendations in the SeSAME package, using the default preprocessing code “QCDPB.” This includes masking low-quality probes, inferring the channel for Infinium-I probes, performing a nonlinear dye bias correction, assessing *P*-value with out of band probes for array hybridization, and noob correction for background fluorescence.

Methylation levels were calculated for each CpG site, represented as beta values. Beta values fall in the range 0 (completely unmethylated) to 1 (completely methylated). Differentially methylated CpG sites were identified between the samples with positive discography scores and the sample with negative discography score. Subsequently, CpG sites associated with promoter regions meeting the minimum criteria of a 5% effect size were analyzed. Sites were self-annotated as promoter-associated using the EPIC v2.0 manifest file and pulling together sites labelled as TSS1550, TSS200, first exon, or 5′UTR (untranslated region) in either the UCSC RefGene or Gencode V41 groups. These sites were then ranked by *P*-value associated with the differential methylation analysis. Top sites with no UCSC RefGene or Gencode V41 gene name were self-annotated using UCSC Genome Browser and chromosome locations provided in the manifest file.

Pathway enrichment analysis of the CpGs associated with promoter sites was run using the methylgsa package in *R*.^36^ Here, all promoter sites (regardless of effect size) and the associated *P*-values from the SeSAME::DML() analysis were run using both the methylglm and methylrra approaches for comparison. Gene ontology, Kyoto Encyclopedia of Genes and Genomes, and Reactome databases were surveyed.

### 2.6. Statistical Analysis

**Figure 1.**
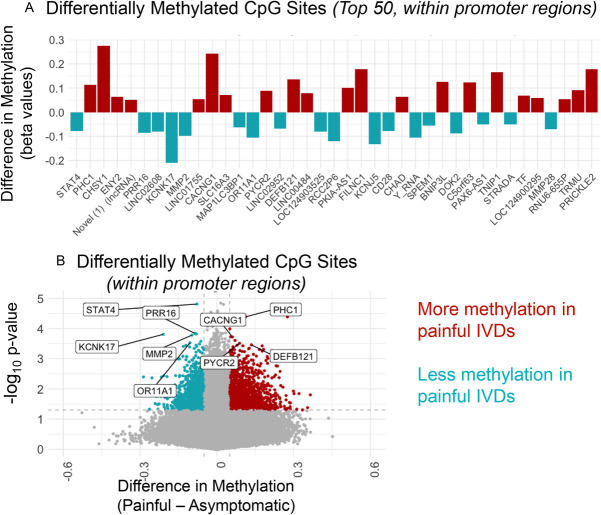
(A) Differences in methylation proportion (β values) between painful and nonpainful promoter associated CpG sites listed by the associated gene name. Top CpG sites were determined by filtering for an effect size of at least 5% then ranking by p-values obtained from differential methylation analysis. Top sites are related to ECM remodeling (MMP2, MMP28, CHSY1, CHAD), immunomodulation (CD28, STAT4), and ion channel function (CACNG1, KNK17). (B) Volcano plot of beta value difference between all painful and nonpainful promoter associated CpG sites; some associated genes are highlighted. Dotted horizonal line = significance cutoff at *P* < 0.05, and dotted vertical lines = beta difference cutoffs of ±0.05. CHAD, chondroadherin; CHSY1, chondroitin sulfate synthase 1; CpG, cytosine guanine dinucleotide.

Given this is a case study with inherent limitations in statistical power, comprehensive statistical analyses were not done. This study's focus, therefore, centers on effect size and rank-order rather than statistical significance. For ranking of differentially methylated sites, the *P*-value between painful and nonpainful IVDs was calculated within the DML() function of the SeSAMe library using *t* test. Enriched pathways were calculated within the methylgsa pipeline, with an adjusted *P* < 0.05 considered significantly enriched. The methylglm function implements logistic regression adjusting for number of probes in enrichment analysis. The methylrra function adjusts multiple *P*-values of each gene by Robust Rank Aggregation.^25^ Here, we used the gene set enrichment analysis (GSEA) preranked approach, ranking by *P*-value obtained from the differential methylation analysis.^40^ Given the limited sample and the unbalanced variance between groups (n = 1 and n = 2), *P*-values were used for rank ordering for GSEA and for inclusion in Figure 1 and Supplemental File 2 (https://doi.org/10.17605/OSF.IO/RE5PG) only. These data are intended for future hypothesis generation.

## 3. Results

### 3.1. Subject information

The subject in this study was a 47-year-old man with discogenic LBP. Outcome measures for the Oswestry Disability Index,^12^ McGill Pain Questionnaire,^30,31^ and Visual Analogue Scale score are listed in Table 1. Information for each IVD, including the MRI grade and discography score, is listed in Table 2. Notably, although all IVDs were similarly degenerated, with MRI grades of 4, one IVD (L5/S1) scored 0/10 during discography test. This contrasts with levels L3/L4 and L4/L5, which scored 10/10 and 8/10, respectively.

**Table 1:** Patient characteristics.

Sex	Male
Age	47
Medication	NSAIDs only, no Tylenol, no opioids
Oswestry Disability Index	50%
McGill Pain Questionnaire	
Total	15
Sensory	12
Affective	3
Visual Analogue Scale (0–100)	30

NSAID, non-steroidal anti-inflammatory drug

**Table 2:** Intervertebral disk characteristics.

Disk	MRI grade	Discography score
L3/L4	4	10/10
L4/L5	4	8/10
L5/S1	4	0/10

### 3.2. Top differentially methylated sites

Beta values (the fraction of total site reads that indicated methylation) were obtained and compared across groups using the SeSAMe pipeline in *R*.^47^ Of more than 935,00 CpG sites, 197,347 sites displayed at least a 5% effect size between painful and nonpainful IVDs, as calculated using the DML function. Filtering for promoter-associated sites (TSS200, TSS1500, exon 1, or 5′UTR) resulted in 57,465 sites. The top 50 of these sites are displayed in Figure 1A, along with the difference in beta value between painful and nonpainful IVDs. Here, a positive value indicates that the painful disks were more methylated at the site compared to the nonpainful disk. For example, a difference score of 0.2 would indicate a 20% increase in methylation in symptomatic IVDs. Figure 1B displays the same information in volcano plot format, with a difference cutoff of 0.05 and *P*-value cutoff of *P* < 0.05. The top 50 sites ranked by *P*-value are displayed; however, all beta values and differential methylation results (*P*-values and effect size) are publicly available in Supplementary File 1 (https://doi.org/10.17605/OSF.IO/RE5PG). Extended information on the top 150 sites, including specific CpG site IDs, *P*-values, and effect sizes, is included in Supplementary File 2 (https://doi.org/10.17605/OSF.IO/RE5PG).

Top promoter region CpGs included genes related to matrix remodeling such as matrix metallopeptidases (MMP2 and MMP28) and chondroitin sulfate synthase 1 (CHSY1). MMP2 and MMP28 both exhibited lower methylation levels in the painful IVDs, while CHSY1 was more methylated in the painful IVDs. Chondroadherin (CHAD) was also more methylated in the painful IVDs. In addition, immunomodulatory genes were identified, such as the T-cell receptor protein CD28 and the transcription factor STAT4; these sites were less methylated in the painful IVD. Furthermore, top differentially methylated promoter CpG sites were found in ion channel related genes, such as Calcium Voltage-Gated Channel Auxiliary Subunit Gamma 1 and Potassium Two Pore Domain Channel Subfamily K Member. Calcium Voltage-Gated Channel Auxiliary Subunit Gamma 1 was more methylated in painful IVDs while Potassium Two Pore Domain Channel Subfamily K Member was less methylated in painful IVD. Moreover, the identification of long noncoding RNAs (lncRNAs) such as LINC02608, LINC01755, and others suggests a regulatory role for noncoding transcripts in painful vs asymptomatic IVD degeneration.

### 3.3. Enrichment analyses

A full list of CpG sites was generated along with *P*-values associated with the differential methylation analysis between painful and nonpainful disks. The methylgsa library in R was used to investigate pathway enrichment using the Gene Ontology, Kyoto Encyclopedia of Genes and Genomes, and Reactome databases; only promoter regions were selected for analysis. Table 3 shows pathways from all analyses with p-adjusted values of *P* ≤ 0.05. Extended data, including the enrichment scores and core enrichment genes, are included in Supplementary File 3 (https://doi.org/10.17605/OSF.IO/RE5PG).

**Table 3:** Enriched pathways between painful and nonpainful intervertebral disks, including the pathway ID, the description of the pathway, the number of genes found in this dataset within the pathway (“size”), the *P*-value and adjusted *P*-value, and the method used to identify the pathways.

ID	Description	Size	*P*	*P*adj	Method
GO:0002312	B-cell activation involved in immune response	105	4.97E-06	0.010	GO:methylglm()
GO:0002285	Lymphocyte activation involved in immune response	247	3.96E-05	0.038	GO:methylglm()
GO:0032526	Response to retinoic acid	105	3.56E-05	0.030	GO:methylrra(GSEA)
GO:0090277	Positive regulation of peptide hormone secretion	106	5.92E-05	0.030	GO:methylrra(GSEA)
GO:0002793	Positive regulation of peptide secretion	108	6.49E-05	0.030	GO:methylrra(GSEA)
GO:0014013	Regulation of gliogenesis	107	8.52E-05	0.030	GO:methylrra(GSEA)
GO:1903532	Positive regulation of secretion by cell	281	9.98E-05	0.030	GO:methylrra(GSEA)
GO:0035270	Endocrine system development	137	1.33E-04	0.030	GO:methylrra(GSEA)
GO:0018209	Peptidyl-serine modification	289	1.53E-04	0.030	GO:methylrra(GSEA)
GO:0045664	Regulation of neuron differentiation	192	1.55E-04	0.030	GO:methylrra(GSEA)
GO:0048732	Gland development	431	1.67E-04	0.030	GO:methylrra(GSEA)
GO:0023061	Signal release	466	2.67E-04	0.037	GO:methylrra(GSEA)
GO:0001501	Skeletal system development	499	2.98E-04	0.037	GO:methylrra(GSEA)
GO:0021782	Glial cell development	119	3.03E-04	0.037	GO:methylrra(GSEA)
GO:0018105	Peptidyl-serine phosphorylation	278	3.58E-04	0.037	GO:methylrra(GSEA)
GO:0090596	Sensory organ morphogenesis	268	3.62E-04	0.037	GO:methylrra(GSEA)
GO:0002790	Peptide secretion	235	3.67E-04	0.037	GO:methylrra(GSEA)
GO:0042445	Hormone metabolic process	226	3.81E-04	0.037	GO:methylrra(GSEA)
GO:0048568	Embryonic organ development	441	3.94E-04	0.037	GO:methylrra(GSEA)
GO:0022839	Monoatomic ion gated channel activity	312	4.12E-04	0.037	GO:methylrra(GSEA)
GO:0022836	Gated channel activity	313	4.49E-04	0.038	GO:methylrra(GSEA)
GO:0042063	Gliogenesis	325	5.36E-04	0.039	GO:methylrra(GSEA)
GO:0060485	Mesenchyme development	308	5.54E-04	0.039	GO:methylrra(GSEA)
GO:0010001	Glial cell differentiation	244	5.63E-04	0.039	GO:methylrra(GSEA)
GO:0034504	Protein localization to nucleus	294	5.63E-04	0.039	GO:methylrra(GSEA)
GO:0021510	Spinal cord development	101	7.19E-04	0.048	GO:methylrra(GSEA)
4010	MAPK signaling pathway	264	1.12E-04	0.004	KEGG:methylrra(GSEA)
5145	Toxoplasmosis	123	2.42E-03	0.036	KEGG:methylrra(GSEA)
4020	Calcium signaling pathway	175	2.79E-03	0.036	KEGG:methylrra(GSEA)
5142	Chagas disease	102	4.13E-03	0.040	KEGG:methylrra(GSEA)
4380	Osteoclast differentiation	119	8.89E-03	0.050	KEGG:methylrra(GSEA)
4062	Chemokine signaling pathway	177	6.88E-03	0.050	KEGG:methylrra(GSEA)

Methods include methylglm(), which implements logistic regression adjusting for number of probes in enrichment analysis and methylrra (GSEA), which adjusts multiple *P*-values of each gene by Robust Rank Aggregation then uses preranked approach to determine pathways.

GSEA, gene set enrichment analysis; GO, gene ontology; KEGG, kyoto encyclopedia of genes and genomes.

Among the enriched pathways, those associated with immune response, such as “B-cell activation involved in immune response” and “lymphocyte activation involved in immune response,” were prominent. Moreover, pathways related to signaling cascades, including the “MAPK (mitogen-activated protein kinase) signaling pathway,” “calcium signaling pathway,” and “chemokine signaling pathway,” were observed. Pathways pertaining to hormonal regulation and secretion, such as “positive regulation of peptide hormone secretion,” “positive regulation of peptide secretion,” and “hormone metabolic process,” were also identified. These findings suggest potential involvement of methylation in regulating the immunomodulatory environment and signaling pathways critical for IVD homeostasis and discogenic LBP.

Cellular development pathways related to the nervous system emerged as significantly enriched, including “regulation of neuron differentiation” and “spinal cord development.” These pathways may imply a role for methylation in regulating neoinnervation of the IVD.

Furthermore, several pathways related to musculoskeletal development and remodeling were identified, such as “mesenchyme development,” “response to retinoic acid,” “osteoclast differentiation,” and “skeletal system development.” These findings suggest a role for methylation in modulating signaling pathways related to structural integrity and remodeling processes within the IVD, thereby contributing to its overall health and function.

No significantly enriched pathways were found for the Reactome database with either method.

## 4. Discussion

This study focused on a single 47-year-old male subject with a history of LBP who underwent surgery. Discography evoked painful symptoms in 2 IVDs, while one IVD with similar degeneration severity by MRI did not evoke painful symptoms with discography. Similar strategies of comparing degenerating vs healthy disks from the same individual or comparing symptomatic vs asymptomatic IVDs have been applied recently to single cell RNA sequencing studies.^10,22^ By exploring the epigenetic landscape of these disks via methylation analysis, we identified multiple differentially methylated CpG sites within the promoter regions of genes and enriched pathways. These findings suggest potential candidate genes for discogenic LBP and underscore the importance of molecular changes, such as altered gene expression secondary to epigenetic reprogramming, in symptomatic vs asymptomatic IVD degeneration.

To understand the role of DNA methylation in the generation of discogenic back pain, differential methylation and pathway analysis was performed comparing 2 painful IVDs to a nonpainful IVD from the same subject. Several enriched pathways point to changes in the IVD microenvironment. For example—“mesenchyme development,” “response to retinoic acid,” “osteoclast differentiation,” and “skeletal system development.” While it is known the microenvironment changes with IVD degeneration, it is interesting that differences in these pathways persist when all disks are equally degenerated, highlighting the potential significance of methylation patterns in painful compared to asymptomatic IVD degeneration.

Promoter CpG sites in genes related to matrix catabolism (MMP2 and MMP28) were less methylated in the painful IVD, suggesting more mRNA and protein expression in painful IVDs. MMP2 correlates with degeneration grade,^37^ so it is interesting that equally degenerated disks displayed different methylation levels of the gene when comparing painful to nonpainful disks. MMP28 is similarly upregulated in degenerated disks.^18^ In contrast, sites related to matrix anabolism (CHSY1, chondroadherin) had higher levels of methylation in the painful IVD, suggesting less transcription and translation to protein. Chondroadherin fragmentation and CHSY1 have been connected to IVD degeneration.^2,20^

NGF is upregulated in painful degenerating IVDs compared to nonpainful IVDs.^14^ However, anti-NGF (nerve growth factor) antibodies have only modest effectiveness in chronic LBP,^34^ suggesting the need to identify other relevant therapeutic targets related to nerve growth and sensitization in chronic LBP. Several significantly enriched pathways were related to neural and glial development, including “regulation of gliogenesis,” “glial cell development,” “regulation of neuron differentiation,” and “spinal cord development.” Several enriched pathways were also related to immune activation in the IVD—“B-cell activation involved in immune response” and “lymphocyte activation involved in immune response.” Many immune cell types, including macrophages, B cells, and T cells, have been connected to IVD degeneration and pain largely through sensitization of nerves via proinflammatory cytokines.^44^ Thus, epigenetic reprogramming of the immune environment and neuroimmune interactions within degenerating IVDs may contribute to discogenic back pain.

Several pathways related to peptide and hormone secretion were significantly enriched “positive regulation of peptide hormone secretion,” “positive regulation of peptide secretion,” and “hormone metabolic process.” Peptide hormones, such as leptin and ghrelin, have been suggested to play a role in IVD degeneration and maintenance, respectively.^27,39^ However, how differential methylation of peptide hormone promoter regions affects the IVD environment and leads to pain is still largely unknown.

Several lncRNA genes were identified in this analysis. These molecules have many regulatory functions at the epigenetic, transcriptional, post-transcriptional, translational, and post-translational levels.^46^ LncRNAs have been implicated in the pathophysiology of several diseases, including IVD degeneration,^45^ and may provide a promising therapeutic target. In the present study, we identified lncRNAs that were either more methylated and less methylated in the painful IVD. While more research is needed to understand the precise roles of specific lncRNAs in IVD degeneration and pain, this finding suggests that DNA methylation may contribute to pathological expression of noncoding RNAs in painful degenerating IVDs, each of which may in turn regulate numerous biological functions.

This study has several limitations. First is the lack of statistical power; in this case study, we utilized 3 disks from one individual. Thus, the goal of this study was inherently exploratory in nature. The use of *P*-values from the differential methylation and enrichment analyses was a pragmatic approach to rank genes and pathways. However, to enhance confidence in these results, fully powered studies involving larger sample sizes of degenerating painful and nonpainful disks should be conducted. Second, the diagnostic accuracy of discography, upon which this study is based, is not universally accepted.^3^ Third, the impact of differential DNA methylation on mRNA and protein expression of individual genes was not investigated here. Future studies should investigate the impact of these genes on IVD degeneration and LBP, for example with genetically engineered models or pharmacologic interventions. Finally, since IVD disease may vary by spinal level, the results of this study may be confounded by the effect of spinal level on DNA methylation in this case study. Future studies with larger sample sizes are needed to account for disk level.

There have been many studies reporting changes in mRNA and protein expression, and a few on DNA methylation^19,21^ in degenerating compared to healthy IVDs, including at the single cell level.^10,22^ Common functional pathways identified in these studies include inflammation (eg, interleukin-1, -6, -8), extracellular matrix (eg, col2a1, aggregan, MMP3), and activation of intracellular signaling cascades. In many studies, it is unknown if the degenerating disks were symptomatic, even when obtained from donors with LBP. Thus, most of what we know to date about painful IVD degeneration reflects the process of degeneration itself rather than the generation of pain. In the current study, 3 disks with similar degeneration severity were included from the same individual, and contrasts were drawn between 2 symptomatic and one asymptotic IVD as assessed by discography. It is, therefore, not surprising that many of the commonly observed genes and pathways associated with IVD degeneration were not identified here.

In conclusion, we present a case study on differentially methylated CpG sites associated with promoter regions of genes between painful and nonpainful degenerated IVDs. Significantly enriched pathways suggest changes in promoter methylation related to extracellular matrix, neuroimmune interactions, and peptide hormone secretion. While the lack of statistical power does not support robust conclusions, this case study offers a list of candidate genes and pathways for further study and provides evidence for a role of DNA methylation in painful IVD degeneration.

## Disclosures

The authors have no conflicts of interest to declare.

This work was supported by National Institutes of Health R21 DA020108 to L.S.S. and L.J.K. (see acknowledgements), R34NS126032 (to D.S. and L.S.S.), and the University of Minnesota Medical School Department of Anesthesiology. The funders had no role in study design, execution, or analysis.

Data statement: The DNA methylation data are available in supplemental table 1 (https://doi.org/10.17605/OSF.IO/RE5PG). Raw FASTQ files will be submitted at the time of acceptance to the NCBI Gene Expression Omnibus (GEO, https://www.ncbi.nlm.nih.gov/geo/) and are also available upon request.

Study pre-registration: Not applicable.

Open materials statement: The components of the research methodology needed to reproduce the reported procedure(s) and analyses are publicly available.

## Appendix A. Supplemental digital content

Supplemental digital content associated with this article can be found online at https://doi.org/10.17605/OSF.IO/RE5PG
